# Association and Agreement of Contact-Based Smartphone Photoplethysmography Compared With Electrocardiography: Protocol for a Systematic Review and Meta-Analysis

**DOI:** 10.2196/84837

**Published:** 2026-02-03

**Authors:** James M Mather, Nicholas Sculthorpe, Ethan Berry, Jacqueline Mair, Nilihan Sanal-Hayes, Lawrence D Hayes

**Affiliations:** 1 Sport and Physical Activity Research Institute, School of Health and Life Sciences, University of the West of Scotland, Glasgow, United Kingdom; 2 Future Health Technologies, Campus for Research Excellence and Technological Enterprise (CREATE), Singapore-ETH Centre, Singapore; 3 Yong Loo Lin School of Medicine, National University of Singapore, Singapore, Singapore; 4 School of Health and Society, University of Salford, Salford, United Kingdom; 5 Lancaster University Medical School, Lancaster University, Health Innovation Campus, Lancaster, LA1 4AT, United Kingdom, 44 07743061291

**Keywords:** photoplethysmography, PPG, mobile, heart rate, validity, artificial intelligence, AI

## Abstract

**Background:**

Mobile health (mHealth), leveraging mobile devices for health measurement and promotion, is rapidly growing. Smartphone cameras can perform photoplethysmography (PPG) to estimate pulse rate (PR) and other features of the cardiac cycle. However, establishing the validity of PR-PPG is essential before it can be adopted for health care applications. There is a pervasive belief that PR-PPG is analogous to heart rate (HR) derived from electrocardiograms (ECGs), and we will conduct a systematic review and meta-analysis to support or challenge this supposition.

**Objective:**

This study aims to synthesize quantitative evidence on the validity of PPG derived from mobile devices (ie, smartphones) for the assessment of HR compared with the gold standard ECG assessment.

**Methods:**

A comprehensive literature search will be performed on CINAHL Ultimate, MEDLINE, ScienceDirect, and Scopus using a predefined search strategy. All retrieved citations will be imported into Rayyan for screening and data management. A minimum of 2 independent reviewers will conduct the title and abstract screening, followed by 2 independent reviewers who will perform full-text screening and data extraction. All stages will be guided by predefined inclusion and exclusion criteria, which will be pilot-tested to ensure consistency and reliability. Any discrepancies will be resolved through discussion with a third reviewer or during a research team meeting. Intrarater reliability will be quantified at the title and abstract stage and the full-text review stage using Cohen κ. To ensure clarity and consistency in the presentation of study characteristics and findings, both narrative synthesis and tabular formats will be used. This review will include studies that report the association and agreement between resting HR and PR from PPG using contact-based smartphone devices versus ECG as the gold standard. PPG signals will be obtained using a contact-based approach, defined as finger-on-camera measurements with the smartphone’s built-in camera and flash. Studies will be excluded if they (1) do not use PPG using contact-based smartphone devices, (2) compare PPG to another collection method other than ECG, or (3) are review articles or case studies.

**Results:**

To inform clinical procedures and future studies, the results will contain data on PR-PPG and HR-ECG association (correlations) and agreement (Bland-Altman analysis), sampling devices, and operating systems. This project is unfunded, and the initial screening is expected to start in the first quarter of 2026, with results anticipated to be published in the first quarter of 2027. The projected timeline for the study includes title and abstract screening from the first quarter of 2026, followed by full-text screening in the second quarter of 2026. Results are anticipated by the third quarter of 2026, with publication expected in the first quarter of 2027. Throughout this period, database searches will be regularly updated to capture any newly published studies meeting the inclusion criteria.

**Conclusions:**

This review will provide a comprehensive understanding of the association and agreement between PR-PPG and HR-ECG. The findings may inform future adoption of PR-PPG and HR-ECG with insights into device or setting characteristics for best agreement or association.

## Introduction

Photoplethysmography (PPG) has been widely used over several decades for the diagnosis, monitoring, and screening of various diseases and disorders, offering clinically relevant physiological insights [1-4]. The term “photoplethysmography” derives from its functional components: “photo” (light), “plethysmo” (volume), and “graphy” (recording) [5]. Initially introduced by Hertzman [4,6] in 1937 to detect blood volume changes, PPG operates by measuring either transmitted (transmissive PPG) or reflected (reflective PPG) light as it interacts with biological tissues [7]. This technique relies on the optical properties of tissue, including absorption, scattering, and transmission [8]. Transmissive PPG detects light that has passed through relatively thin tissue regions, such as the fingers, toes, or earlobes. In contrast, reflective PPG captures light that is scattered back from the skin, which results in a reduction in detected light intensity [9]. While transmissive PPG generally provides more stable signal quality [10], reflective PPG offers greater versatility in terms of measurement site, enabling its application in anatomical regions such as the forehead, wrist, carotid artery, and esophagus—locations where transmissive PPG is less feasible [11-14].

PPG operates based on the Beer-Lambert law, which describes the attenuation of light intensity as a function of the extinction coefficient, concentration of the absorbing medium, and the optical path length through which light travels [15]. Leveraging this principle, a range of PPG devices are used in clinical settings to measure physiological parameters such as pulse rate (PR), a key vital sign [7]. Clinically, PPG is commonly used to monitor cardiac-induced fluctuations in blood volume within microvascular beds at peripheral anatomical sites including the finger, forehead, earlobe, and toe [16,17].

Since the introduction of the first iPhone in 2007, smartphones have become ubiquitous worldwide and are increasingly recognized as practical tools for data collection, addressing several limitations inherent in traditional methods [18]. Conventional health monitoring typically involves periodic, scheduled clinical visits, which may fail to capture dynamic physiological changes that occur longitudinally or during routine daily activities [3,19,20]. In this context, smartphones equipped with integrated cameras offer a cost-effective alternative for PPG acquisition, eliminating the need for additional external devices such as wearables [21].

Consequently, smartphone-based PPG has the potential to extend access to underserved populations, particularly those facing demographic, geographic, or socioeconomic barriers to health care access and delivery [22-24]. The adoption of mobile health (mHealth) technologies has accelerated in recent years, particularly following the COVID-19 pandemic, which underscored the utility of remote and prospective health and symptom monitoring [21,25-27]. As such, the increasing prevalence of smartphone-enabled telemedicine is likely to persist and may play a significant role in advancing global health equity, aligning with targets outlined in the United Nations Sustainable Development Goals, specifically Sustainable Development Goal 3: Good Health and Well-being [28,29].

Smartphone-based PPG can estimate resting PR through the measurement of distal pulse signals at rest, as well as during other activities such as exercise or cognitive tasks [7]. While smartphone-based PPG can measure resting PR through peripheral pulse detection, accuracy hinges on proprietary algorithms that are often undisclosed in the literature. The lack of algorithmic transparency can hinder reproducibility and trust in the results. This is particularly problematic given the proliferation of mHealth technologies and the resulting necessity that such tools be reliable and valid when compared with gold standard measurements before widespread adoption [30]. An earlier meta-analysis by De Ridder et al [31] showed that smartphone PPG could yield results consistent with electrocardiogram (ECG), pulse oximetry, and radial pulse measurements. However, they identified significant variability due to sample characteristics, environmental conditions, and the diversity of smartphone hardware. Their review also reflected on the outdated technology assessed at the time, such as iPhone 5 and Galaxy S4 models. Subsequent technological advancements, including higher-resolution cameras and improved sensors, warrant an updated synthesis of the evidence [21]. Our recent scoping review [3] identified 10 studies directly comparing PR derived from smartphone-based PPG (PR-PPG) and heart rate (HR) derived from ECG (HR-ECG) but did not quantify their agreement or association. This protocol aims to address that gap by conducting a rigorous meta-analysis of studies evaluating the association and agreement between smartphone-based PR-PPG and HR-ECG.

The primary research question is as follows: what is the level of association and agreement between PR-PPG and HR-ECG at rest?

Secondary research questions include the following:

- Does device type or operating system (eg, iOS vs Android) influence the association and agreement of PR-PPG?
- What is the methodological quality and risk of bias among included studies?

We hypothesize that smartphone-derived PR-PPG may provide a valid alternative to HR-ECG at rest. Nonetheless, the current evidence base is likely to be limited and methodologically heterogeneous due to variations in device specifications, study design, and participant characteristics.

## Methods

### Eligibility Criteria

The eligibility criteria will be assessed using the PICO (population, intervention, comparison, and outcome) framework. For the population component, we will only accept studies that involve humans. For the intervention component, as this is an analysis of association and agreement, rather than magnitude of change, there will be no intervention per se. However, this systematic review will investigate studies that measure PR-PPG via front- or rear-facing camera of a smartphone using a contact-based approach and HR-ECG. Contact-based smartphone PPG is defined as a finger-on-camera measurement using the device’s built-in camera and flash. Participants place the fingertip over the camera lens while the flash illuminates the skin to capture PPG signals. Noncontact methods, such as facial video PPG, are not included in this study. We will restrict inclusion to studies that report agreement or correlation, and we will not compute association or agreement from summary data. For the comparison component, the inclusion of a control group is not necessary in this analysis. Regarding outcome measures, resting pulse rate (beats per min [bpm]) from PR-PPG and resting HR (bpm) from HR-ECG will be reported in the original studies. Moreover, to quantify association and agreement, studies must report a correlation coefficient (Pearson correlation coefficient, concordance correlation coefficient, or interclass correlation coefficient) or a Bland-Altman analysis between PR-PPG and HR-ECG.

The following exclusion criteria will apply: studies using external devices (eg, medical sensors or wearables) connected to smartphones for data acquisition and papers that do not assess the validity of PR-PPG against HR-ECG as an outcome measure. Only original research articles presenting at least preliminary quantitative findings will be included. Qualitative studies, case reports, and literature reviews will not be eligible for inclusion in this review.

### Search Strategy

This meta-analysis will be performed in accordance with the PRISMA (Preferred Reporting Items for Systematic Reviews and Meta-Analyses) guidelines. The study protocol has been registered on the Open Science Framework (OSF). Any deviations from this protocol during the review will be documented with justifications and time stamps to ensure full transparency, and database searches will be updated close to the time of analysis.

A comprehensive search strategy has been developed to strike a balance between thorough coverage and practical scope, ensuring methodological rigor. Five electronic databases will be searched: CINAHL Ultimate, MEDLINE, ScienceDirect, Scopus, and EMBASE. These databases were selected for their relevance across health sciences, biomedical research, signal processing, and behavioral science. CINAHL Ultimate is included for its strong representation of allied health literature. MEDLINE, accessed via EBSCOhost, provides authoritative sources in biomedical and cardiovascular research. ScienceDirect is chosen for its wide range of journals covering computational and physiological aspects of PPG. Scopus is used for its comprehensive indexing and citation tracking capabilities. Embase is searched because it offers broader, deeper, and more clinically oriented coverage than many other databases, especially for medical, pharmaceutical, and health-related research. In addition to peer-reviewed literature, gray literature will be searched. This includes preprint servers (eg, MedRxiv and arXiv), theses and dissertations (ProQuest), conference proceedings (eg, Web of Science), and institutional repositories such as DSpace. Citation chaining will also be used by examining reference lists and citing articles (via Google Scholar and Crossref), along with CoCites to identify related work through citation patterns. The search strategy will be refined iteratively if it produces excessive irrelevant records, with adjustments made to keyword combinations, Boolean logic, and subject headings. Known relevant studies will be used to test the sensitivity and specificity of the search. If required data are missing or unclear, the corresponding authors will be contacted using a standardized template. Follow-up emails will be sent 2 and 4 weeks after the initial message if no reply is received. All correspondence will be logged and summarized in the manuscript and supplementary materials. A final search update will be conducted before manuscript submission if more than 6 months have passed since the initial search.

### Search Terms

The following is an example Boolean search string used for Scopus: TITLE-ABS-KEY (“heart rate” OR hr) AND TITLE-ABS-KEY (photoplethysmography OR ppg OR “remote ppg” OR “camera-based ppg” OR “remote photoplethysmography” OR “camera based photoplethysmography”) AND TITLE-ABS-KEY (smartphone* OR “mobile phone” OR “mobile device” OR camera* OR “smartphone photoplethysmography” OR “phone-based ppg” OR “smartphone ppg”) AND TITLE-ABS-KEY (electrocardiogram OR ecg OR ekg OR electrocardiography) AND TITLE-ABS-KEY (validity OR valid OR accuracy OR agreement OR correlation OR reliability).

The example search string includes Scopus syntax; these terms and syntax will be adapted for all databases planned for inclusion in the search. The search period will begin in 2007 to ensure inclusion of early studies related to the first smartphone models (eg, iPhone released in 2007) and will extend through the end of 2025. The review will focus on correlation coefficients comparing HR estimates from PPG to ECG. Supplementary data will include study setting, participant demographics, smartphone model, app characteristics, sampling frequency, camera specifications, ECG details, and environmental conditions during data collection.

### Study and Source of Evidence Selection

All references retrieved from the database search will be imported into Rayyan (Rayyan Systems Inc) [32], where duplicate entries will be automatically identified and removed. The screening process will take place in two stages: (1) title and abstract screening and (2) full-text screening.

In both stages, 2 independent reviewers will apply *a priori* inclusion and exclusion criteria. Reviewers will work in a blinded manner, with Rayyan’s platform enabling independent decisions without revealing each other’s judgments. Disagreements will be flagged automatically and resolved through discussion. If consensus cannot be achieved, a third reviewer will serve as an adjudicator.

To improve consistency, a calibration phase will be conducted before formal screening. Reviewers will test the inclusion and exclusion criteria on a sample of records to align interpretation and application. Interrater reliability will be evaluated using Cohen κ statistic, which measures agreement beyond chance. This metric will be calculated at both the abstract and full-text screening phases. Screening decisions and bibliographic metadata will be exported in RIS and CSV formats and archived in an open-access repository (eg, OSF) at the time of submission or acceptance.

### Data Extraction

Before full-scale data extraction, a pilot phase will be conducted where all reviewers independently extract data from a small subset of studies (5%‐10%). This exercise will be used to refine the extraction template and standardize the approach.

In the main extraction phase, 2 reviewers will independently extract data using a structured and pretested form. Extracted variables will include correlation coefficients and mean bias and limits of agreement between PR-PPG and HR-ECG, participant demographics, smartphone specifications, ECG characteristics, and data collection procedures. As we will include correlation coefficients and Bland-Altman analysis, these tests will report both *r* values (−1 to 1) and agreement values (in bpm or Hz). Therefore, it is important to outline our harmonization strategy. Where a study reports HR or PR in Hz, we will apply the following formula:


$$\text{Hz}=\frac{\text{bpm}}{60}$$

During the risk of bias assessment, the Quality Assessment of Diagnostic Accuracy Studies (QUADAS-2) will be used independently by 2 reviewers. QUADAS-2 evaluates 4 domains: patient selection, index test, reference standard, and flow and timing. Each domain will be rated as low, high, or unclear risk of bias, and applicability concerns will be assessed for patient selection, index test, and reference standard.

Disagreements will be resolved through consensus or by involving a third adjudicator. Any ambiguities in reported data will be flagged for discussion. Missing values will be marked as “NR” (not reported), and no estimations will be made unless explicitly stated. All data entries will be double-checked by a second reviewer for accuracy. Where appropriate, artificial intelligence–assisted tools may be used to support metadata extraction or to highlight relevant text, but all final data entries will be manually reviewed and verified. Reconciliation logs and resolution steps will be documented throughout the process. Data collection will adhere to PRISMA and Cochrane Handbook guidelines. Finalized data will be formatted for meta-analysis and saved using a standardized file naming convention. These files will also be uploaded to the OSF repository.

Details on each included study will be systematically documented, beginning with key bibliographic information, such as the study title, authors, publication year, journal name, and study setting (eg, academic or clinical environments). Further extracted information will include total sample size; participant demographics (age, sex, health status, and skin pigmentation); country of study; smartphone model and specifications (eg, camera resolution and flash use); application name and whether it is a proprietary commercial application, open-source software, or custom-developed code; sampling rate of the PPG signal; camera orientation used (front- vs rear-facing); channel used in data computation; ECG equipment used; electrode placement details; ECG processing methods, environmental conditions, and participant preparation (eg, posture, breathing instructions, and dietary control); duration of recording; and number of measurement trials.

In addition to the extracted information above, the extraction form will capture relevant statistical outcomes, such as correlation coefficients, CIs, mean bias, limits of agreement, and any additional reported metrics that relate to association or agreement. Risk of bias for each study will be evaluated using the QUADAS-2 tool, with domain-specific judgments recorded and justified.

To ensure data accuracy, each extraction will be independently performed by 2 reviewers. Their entries will be compared side by side, with discrepancies resolved through consensus. If a resolution cannot be reached, a third reviewer will arbitrate. All final decisions and justifications will be recorded in a reconciliation log. Each data extraction file will follow a standardized naming convention (eg, StudyID_Initials_Extraction.xlsx) and be uploaded to the OSF repository upon completion. Once finalized, the cleaned and verified dataset will be formatted for meta-analysis and made available for public access alongside the manuscript, unless restricted by publisher guidelines.

### Data Analysis and Presentation

The analysis will use the Fisher r-to-z transformed correlation coefficient as the primary outcome measure of association. The analysis will use the pooled mean difference and limits of agreement as the primary outcome measure of agreement. A random-effects model will be applied to the data, with all analyses performed in RStudio (Posit PBC) using the *metafor* package. Welz et al [33] proposed that when conducting a meta-analysis involving Fisher r-to-z transformed correlation coefficients, especially in the presence of study heterogeneity, using a random-effects model with appropriate variance estimation techniques yields more reliable and generalizable results compared with fixed-effects models. Therefore, random-effects models will be used. Subgroup analyses will only be performed when there are at least 5 studies per subgroup. Metaregression will require a minimum of 10 studies per covariate to reduce risk of overfitting. Tests for funnel plot asymmetry will only be conducted when the meta-analysis includes 10 or more studies, as recommended by the Cochrane Handbook. Sensitivity analyses will be interpreted cautiously when the evidence base is small.

To identify potential outliers and influential studies, studentized residuals and Cook distances will be examined [34]. Studies with studentized residuals exceeding the 100×(1–0.05/(2×k))th percentile of the standard normal distribution (applying a Bonferroni correction with 2-sided *α*=.05 for k studies) will be flagged as potential outliers. Studies with Cook distances greater than the median plus 6 times the IQR of Cook distances will be deemed influential. Outliers and influential studies will be retained in the primary analysis but flagged for sensitivity analyses to assess their impact on pooled estimates. Funnel plot asymmetry will be assessed using the rank correlation test [35] and regression test [36], where the SE of observed outcomes serves as the predictor. Heterogeneity will be evaluated through *I*², Cochran *Q*, and τ^2^ statistics. Where possible, subgroup analyses will explore variability in PR-PPG validity based on factors such as operating system, participant characteristics (eg, age and sex), and study design.

Moderator analyses will investigate the influence of PPG parameters and participant traits on outcomes, while sensitivity analyses will test the effects of risk of bias. Additional sensitivity checks will consider the impact of excluding high-risk studies, approaches to missing data, and methodological quality. Provided sufficient data, metaregression will examine relationships between continuous variables and effect sizes. Results will be displayed using forest and funnel plots. Conclusions concerning association will be based on effect sizes, with overall effect sizes of *r*>0.9 (approximately z>1.5) considered valid. For context, *r*=0.70 is typically regarded as very large and approximately corresponds to z=0.8. Conclusions concerning agreement will be based on bias and limits of agreement (LoA). Bias <1 bpm and LoA <5 bpm will be considered excellent agreement and acceptable for medical-grade use, bias <3 bpm and LoA <10 bpm will be considered acceptable for general PR monitoring and may be borderline for medical-grade use, and bias >5 bpm or LoA >10 bpm will be considered poor agreement and not clinically interchangeable. High heterogeneity (*I*²>50%) will warrant cautious interpretation and further exploration through subgroup or moderator analyses. Confidence in conclusions will be guided by the QUADAS-2 framework depending on the quality of accuracy.

## Results

To inform clinical procedures and future studies, the results will contain data on PR-PPG and HR-ECG association (correlations) and agreement (Bland-Altman analysis), sampling devices, and operating systems. This project is unfunded, and the initial screening is expected to start in the first quarter of 2026, with results anticipated to be published in the first quarter of 2027. Throughout this period, database searches will be regularly updated to capture any newly published studies meeting the inclusion criteria.

**Figure 1.**
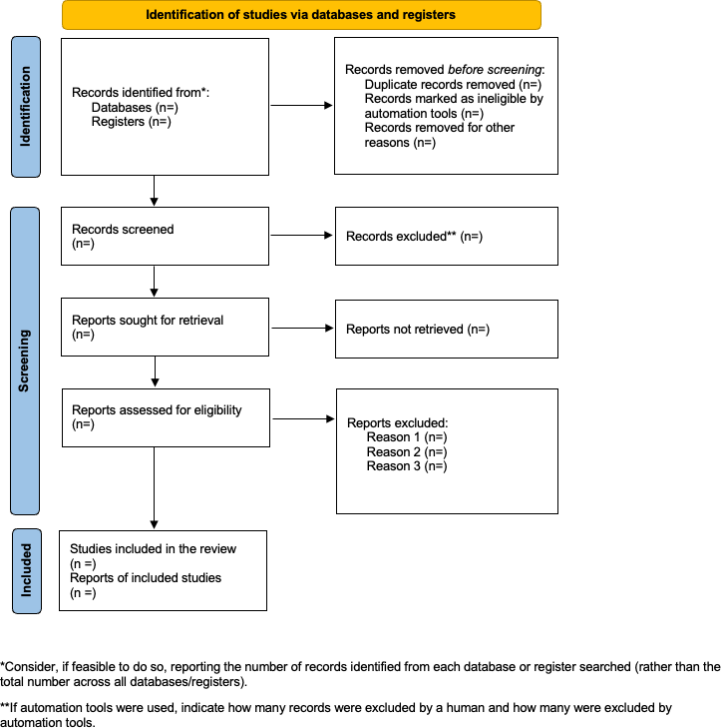
The PRISMA (Preferred Reporting Items for Systematic Reviews and Meta-Analyses) flow diagram visually summarizes the screening process.

A summary of the study selection procedure is presented in Figure 1 to ensure transparency during study selection. Reporting will follow the PRISMA checklist (Checklist 1).

## Discussion

This systematic review and meta-analysis aims to be the first to provide an updated quantification of the association and agreement of PR-PPG compared with HR-ECG at rest. The rapid development in technology requires continuous assessment of validity, and this meta-analysis will provide important findings, indicating whether PR-PPG can be used with confidence. There is a pervasive belief that PR-PPG is analogous to HR-ECG, and herein we will provide an updated systematic review and meta-analysis to support or challenge this supposition. We hypothesize that smartphone-based PR-PPG will demonstrate strong association and acceptable agreement with HR-ECG at rest. These findings would support the potential of PR-PPG as a valid alternative for remote monitoring in clinical and nonclinical settings.

The decision to undertake a meta-analysis was driven by the need to evaluate the level of agreement between PR-PPG and HR-ECG, including modifying parameters such as frame rate, camera location, skin pigmentation, and operating system. Moreover, if there is a lack of knowledge regarding modifying variables, this systematic review will provide opportunities for future research. By providing context to current findings, this research can guide future studies concerning PR-PPG and its application. Previous reviews, such as the one by De Ridder et al [31], indicated that smartphone-based PR-PPG could approximate HR-ECG. However, these analyses were constrained by outdated technology and lacked detailed agreement metrics, limiting their clinical applicability. The meta-analysis by De Ridder et al [31] included studies published between 2009 and 2016, which means smartphone models assessed were from that era. These typically included early-generation devices such as the Apple iPhone 4 and iPhone 5 series (released in 2010 and 2012), the Samsung Galaxy S3 and S4 (released in 2012 and 2013), and other similar Android models from that period. These devices are now 10 to 15 years old, and their hardware (camera resolution, LED flash quality, and processing power) is significantly outdated compared to current smartphones. This is why the findings from the study by De Ridder et al [31], while useful at the time, may not fully reflect the performance of modern devices with advanced sensors and algorithms. Our meta-analysis aims to address these gaps by including studies that use modern smartphones and operating systems, reflecting current technological capabilities. By quantifying agreement using Bland-Altman analysis alongside correlation measures, we will provide a more robust assessment of interchangeability. Moreover, by exploring potential moderators, such as device type, operating system, and participant demographics, we will be able to better understand sources of variability.

This review has several strengths, including a rigorous methodology following PRISMA-P (Preferred Reporting Items for Systematic Review and Meta-Analysis Protocols) guidelines and preregistration, clear eligibility criteria using the PICO framework, a comprehensive search strategy of multiple databases, robust screening and data extraction with dual independent reviewers with calibration and Cohen κ for reliability, and the use of structured extraction forms and QUADAS-2 for bias assessment. Our analysis plan includes random-effects meta-analysis using Fisher r-to-z transformation, outlier diagnostics, funnel plot asymmetry tests, and moderator analyses. The main limitations are those of the original studies. These include potential ambiguity in reporting, heterogeneity in study design and reporting, selective reporting, and lack of algorithm transparency due to proprietary software. The results from this systematic review and meta-analysis could identify areas for future research, such as lack of diversity in participants, potential for integration with telehealth and electronic health records, and device-specific or operating system–specific optimization.

## Supplementary material

10.2196/84837 Checklist 1 PRISMA checklist.

## Abbreviations

AI: artificial intelligence
ECG: electrocardiogram
HR: heart rate
mHealth: mobile health
OSF: Open Science Framework
PICO: population, intervention, comparison, and outcome
PPG: photoplethysmography
PR: pulse rate
PRISMA: Preferred Reporting Items for Systematic Reviews and Meta-Analyses
PRISMA-P: Preferred Reporting Items for Systematic Review and Meta-Analysis Protocols
QUADAS-2: Quality Assessment of Diagnostic Accuracy Studies
